# Changes in Emergency Department Visits for Cannabis Hyperemesis Syndrome Following Recreational Cannabis Legalization and Subsequent Commercialization in Ontario, Canada

**DOI:** 10.1001/jamanetworkopen.2022.31937

**Published:** 2022-09-16

**Authors:** Daniel Thomas Myran, Rhiannon Roberts, Michael Pugliese, Monica Taljaard, Peter Tanuseputro, Rosalie Liccardo Pacula

**Affiliations:** 1 Clinical Epidemiology Program, Ottawa Hospital Research Institute, Ottawa, Ontario, Canada; 2 Department of Family Medicine, University of Ottawa, Ottawa, Ontario, Canada; 3 ICES Ottawa, Ottawa Hospital Research Institute, Ottawa, Ontario, Canada; 4 School of Epidemiology and Public Health, University of Ottawa, Ottawa, Ontario, Canada; 5 Bruyère Research Institute, Ottawa, Ontario, Canada; 6 Department of Medicine, University of Ottawa, Ottawa, Ontario, Canada; 7 Sol Price School of Public Policy, Schaeffer Center for Health Policy & Economics, University of Southern California, Los Angeles

## Abstract

**Importance:**

Prior research suggests that the legalization of recreational cannabis is associated with increases in cannabis hyperemesis syndrome (CHS), but it is unclear how cannabis commercialization (ie, greater retail store access as well as increased variety and potency of cannabis products) may be associated with these changes.

**Objectives:**

To examine changes in the number and characteristics of CHS emergency department (ED) visits from before to after legalization of cannabis in Ontario, Canada.

**Design, Setting, and Participants:**

This repeated cross-sectional study used interrupted time-series analyses to examine immediate and gradual changes in ED visits for CHS in Ontario, Canada, during 3 time periods: prelegalization (January 2014-September 2018), legalization with product and retail store restrictions (October 2018-February 2020), and commercialization with new products and expanded stores, which coincided with the COVID-19 pandemic (March 2020-June 2021). Data were obtained from routinely collected and linked health administrative databases. All individuals aged at least 15 years and who were eligible for Ontario’s Universal Health Coverage were included. Data were analyzed between March and July 2022.

**Main Outcomes and Measures:**

Monthly counts of ED visits for CHS per capita.

**Results:**

There were 12 866 ED visits for CHS from 8140 individuals during the study. Overall, the mean (SD) age was 27.4 (10.5) years, with 2834 individuals (34.8%) aged 19 to 24 years, 4163 (51.5%) females, and 1353 individuals (16.6%) with a mental health ED visit or hospitalization in the 2 years before their first CHS ED visit. Nearly 10% of visits (1135 visits [8.8%]) led to hospital admissions. Monthly rates of CHS ED visits increased 13-fold during the 7.5-year study period, from 0.26 visits per 100 000 population in January 2014 to 3.43 visits per 100 000 population in June 2021. Legalization was not associated with an immediate or gradual change in rates of ED visits for CHS; however, commercialization during the COVID-19 pandemic period was associated with an immediate increase in rates of CHS ED visits (incidence rate ratio [IRR], 1.49; 95% CI, 1.31-1.70). During commercialization, rates of CHS ED visits increased more in women (IRR, 1.49; 95% CI, 1.16-1.92) and individuals older than the legal age of cannabis purchase (eg, age 19-24 years: IRR, 1.60; 95% CI, 1.19-2.16) than men (IRR, 1.08; 95% CI, 0.85-1.37) and individuals younger than the legal age of purchase (IRR, 0.78; 95% CI, 0.42-1.45).

**Conclusions and Relevance:**

This cross-sectional study found large increases in CHS ED visits during the period of time when the market commercialized and the COVID-19 pandemic occurred. Greater awareness of CHS symptoms by ED staff in regions where legal commercialized cannabis markets exist is indicated.

## Key Points

**Question:**

Have emergency department (ED) visits for cannabis hyperemesis syndrome (CHS) changed after cannabis legalization and the subsequent commercialization (ie, store and product expansion) of the cannabis retail market in Ontario, Canada?

**Findings:**

In this cross-sectional study including 12 866 ED visits for CHS from 8140 individuals, rates of CHS ED visits increased by 13-fold over 7.5 years. Legalization was not associated with increased CHS visits, but market commercialization, which overlapped with the COVID-19 pandemic, was.

**Meaning:**

These findings suggest that commercialization of cannabis markets may be a driving factor of increased CHS ED visits in Ontario; therefore, health care practitioners should be aware of CHS symptoms and treatment.

## Teaser

This cross-sectional study examines changes in the number and characteristics of emergency department (ED) visits for cannabis hyperemesis syndrome from before to after legalization and commercialization of cannabis in Ontario, Canada.

## Introduction

Cannabis is one of the most commonly used substances in Canada and the United States: in 2019, 17.5% of individuals in the US and 21.0% of individuals in Canada reported using cannabis in the past year.^1,2^ There is increasing recognition that regular cannabis use is associated with various health concerns, including psychosis, anxiety, depression, altered brain functioning, and dependence.^3,4,5,6^ Cannabis hyperemesis syndrome (CHS) is a lesser-known complication associated with persistent use of high potency cannabis, involving repeated episodes of nausea, vomiting, and abdominal pain.^7,8^ To date, several studies have found increases in emergency department (ED) visits owing to vomiting or CHS after the legalization of medical or recreational cannabis.^9,10,11,12^ However, the characteristics of individuals with CHS have only previously been described in small studies or case series, limiting our understanding of who is at risk of this condition. In addition, there is limited information on how recreational cannabis legalization and different regulatory policies (eg, access to retail stores and product types) are associated with the frequency and severity of CHS or the clinical characteristics of individuals with CHS.

This study addressed these important gaps by leveraging individual-level health administrative data for all residents in Ontario (Canada’s most populous province) and a natural experiment resulting from the legalization of recreational cannabis. On October 17, 2018, Canada became the second country worldwide to legalize the sale and use of recreational or nonmedical cannabis for adults.^13^ Each province in Canada was allowed to set its own rules regarding retail sales, including the legal age of purchase (19 years in Ontario), store access, and product availability. In Ontario, for the first 6 months after legalization, sales were restricted to a government-operated website.^14^ Between April 2018 and March 2020, only 62 stores were permitted to open (0.5 stores per 100 000 individuals aged ≥15 years). In April 2020, Ontario lifted the cap on retail stores. Between April 2020 and June 2021, the number of retail stores per capita increased 13-fold (826 stores or 6.6 stores per 100 000 individuals aged ≥15 years), and monthly cannabis sales in Ontario increased 3-fold ($3.2 CAD [US $2.5] per individual aged ≥15 years in April 2020 to $9.6 CAD [US $7.5] per individual aged ≥15 years in June 2021).^15^ In addition, during the first 15 months after legalization, only dried cannabis flowers and oils could be sold. Starting in January 2020, Ontario permitted the sale of a wider variety of cannabis products with higher tetrahydrocannabinol (THC) potency, including concentrates, THC-infused beverages, and commercially produced edibles. We previously found substantial differences in changes in cannabis-related ED visits (eg, intoxication and dependence) by regulatory period.^16^ During the initial period of restricted stores and products, there was little change in cannabis-related ED visits.^16^ However, visits increased substantially starting in early 2020, coinciding with the maturing cannabis retail market and the COVID-19 pandemic.^16^

The objective of this study was to evaluate changes in CHS ED visits after legalization, specifically, their frequency, severity, and individual characteristics. We hypothesized that the initial period after legalization (limited retail market, product potency, and variety) would not be associated with a substantial increase in CHS ED visits. However, we expected to see increased CHS ED visits during the period of market expansion (stores and products). The period of market expansion overlapped with the COVID-19 pandemic, during which ED use declined, and there have been concerns of negative mental health outcomes associated with the pandemic.^17,18^ To account for potential pandemic influences on mental health and overall health service use, we examined rates of CHS ED visits as a share of total ED visits for any cause, as well as a share of ED visits for vomiting, mental health, or substance use. In addition, to better understand how outcomes associated with legalization may affect populations unevenly, we explored changes in CHS ED visits for subgroups by age (including older than and younger than the legal age of purchase), sex, and neighborhood income.

## Methods

The use of the data in this cross-sectional study was authorized under section 45 of Ontario’s Personal Health Information Protection Act (PHIPA) and did not require review by a research ethics board or informed consent. This study followed the Strengthening the Reporting of Observational Studies in Epidemiology (STROBE) reporting guideline.

### Study Design, Population, and Data Sources

We conducted a repeated cross-sectional population-level study using health administrative databases for Ontario, Canada. We included all Ontario residents aged 15 to 105 years who were eligible for the province’s single-payer universal health insurance between January 2014 and June 2021 (14 375 697 individuals). We obtained data from 6 individual-level databases (containing different types of health care visits and individual demographics), which were linked using unique coded identifiers and analyzed at ICES (eMethods in the Supplement). We used an interrupted time-series (ITS) method with segmented regression analysis to assess the immediate and gradual changes in CHS ED visits associated with legalization and commercialization.

### Exposures

We divided our study into 3 periods: before legalization (January 2014-September 2018), after legalization but with restricted retail stores and cannabis products (hereafter, *legalization*) (October 2018-February 2020), and after legalization and with unlimited retail stores and expanded products (hereafter, *commercialization*), which overlapped with the COVID-19 pandemic (March 2020-May 2021).

### Outcomes

Our primary outcome was the monthly rate of ED visits owing to CHS per 100 000 individuals. Because there is no diagnostic code for CHS, we followed the previous literature identifying CHS ED visits as those in which vomiting (*International Statistical Classification of Diseases and Related Health Problems, Tenth Revision, Canada* [*ICD-10-CA*] code R11) was the primary diagnosis and a cannabis harm (*ICD-10-CA* code F12 or T40.7) was an additional diagnosis.^19,20^ Since CHS is not widely recognized, we developed a secondary outcome measure termed *sensitive CHS ED visits*, which includes the primary outcome definition and an ED visit with a primary diagnosis of vomiting (*ICD-10-CA* code R11) plus an ED visit owing to a cannabis harm in the 6 months preceding or following the incident vomiting visit.^19,20^ We constructed 4 additional outcome measures to better isolate the association with commercialization vs with the COVID-19 pandemic. These outcomes normalized our CHS ED visits by total ED visits for any cause and for vomiting, mental health, or substance use.^21^

We characterized the clinical course of CHS ED visits through 3 measures. We examined the triage acuity of visits using the Canadian Trauma and Acuity Scale, which is a validated ordinal score used to classify the acuity of ED presentations ranging from 1 (resuscitation) to 5 (nonurgent).^22^ We examined the proportion of CHS ED visits resulting in hospitalization and the proportion of CHS ED visits with a repeat CHS ED visit within 6 months.

### Additional Covariates

We identified prior mental health service use over the past 2 years of individuals with CHS ED visits, including outpatient visits, ED visits, and hospitalizations.^21^ We classified individuals as living in urban or rural regions and neighborhood income groups (categorized into quintiles) using Statistics Canada census data.^23^

### Statistical Analysis

We present descriptive statistics to characterize and compare individuals with ED visits owing to CHS across the 3 study periods. Individual characteristics reflect those recorded at the first ED visit in each policy period or those recorded at the time of the first visit for the full study period.

We used segmented Poisson regression analysis to examine changes in ED visits for CHS over time. The dependent variable was the monthly count of CHS ED visits with the natural log of the desired denominator (primary outcome: population at-risk; secondary outcome: all-cause visits) included as an offset. Our secondary outcomes were included to consider bias in changes in overall ED use and mental health and substance-related ED use associated with pandemic stressors. We included a slope and level change corresponding to both legalization and commercialization to examine immediate and gradual changes in visits. We included indicators representing the 4 seasons to account for seasonal variation, and all analyses included first-order autocorrelation. When examining our primary outcome (per capita rates of CHS ED visits), we included an indicator variable for March and April 2020 to account for large declines in overall ED use during the first 2 months of the pandemic. The immediate and gradual changes of each interruption were expressed as incidence rate ratios (IRRs) with 95% CIs.

We estimated combined immediate and gradual changes by comparing model-based estimates 17 months after legalization (February 2020) to the projected secular trend from the prelegalization period (ie, the estimate that would have been observed had legalization not occurred)^23^ and model-based estimate 16 months after commercialization and the start of the COVID-19 pandemic (June 2021) to the projected secular trend from the postlegalization period (ie, the estimate that would have been observed had commercialization and the pandemic not occurred).^24^ We ran stratified models comparing changes in our primary outcome over the 3 periods for men vs women; individuals aged 15 to 18 years, 19 to 24 years, 25 to 44 years, and 45 years or older; and individuals living in the highest vs lowest neighborhood income quintile.

All statistical analyses were completed using in SAS Enterprise Guide version 7.1 (SAS Institute). Statistical significance was determined by 95% CIs that did not cross 1. Data were analyzed between May and July 2022.

## Results

Overall, our study included 8140 individuals with 1 or more ED visits owing to CHS between January 2014 and June 2021. The mean (SD) age was 27.4 (10.5) years, with 2834 individuals (34.8%) aged 19 to 24 years, and 4163 individuals (51.1%) were female. More individuals lived in neighborhoods in the poorest income quintile in Ontario (2524 individuals [31.0%]) compared with the richest income quintile (1303 individuals [16.0%]). Of 8140 individuals included, 1990 individuals (24.5%) had an ED visit or hospitalization for a mental health condition (1353 individuals [16.6%]) or substance use (960 individuals [11.8%]) in the past 2 years. One-quarter of individuals (2109 individuals [25.9%]) had an ED visit for vomiting in the 6 months before their first ED visit for CHS. Across the 3 periods, the mean age of individuals with CHS and their neighborhood’s socioeconomic status were relatively stable. However, the proportion of visits by females increased from 1439 females (47.5%) before legalization to 2036 females (53.5%) during the commercialization period (Table 1).

**Table 1.** Characteristics of Ontario, Canada, Residents With at Least 1 ED Visit for Cannabis Hyperemesis Syndrome During the Study

Characteristic	Period, No. (%)^a^			
	Prelegalization (n = 3028)^b^	Legalization (n = 2349)^c^	Commercialization (n = 3804)^d^	Overall (N = 8140)
Sex				
Female	1439 (47.5)	1175 (50.0)	2036 (53.5)	4163 (51.1)
Male	1589 (52.5)	1174 (50.0)	1768 (46.5)	3977 (48.9)
Age, y				
Mean (SD)	27.4 (10.5)	28.2 (10.9)	28.8 (10.8)	28.1 (11.0)
15-18	446 (14.7)	315 (13.4)	408 (10.7)	1117 (13.7)
19-24	1102 (36.4)	807 (34.4)	1264 (33.2)	2834 (34.8)
25-44	1232 (40.7)	994 (42.3)	1780 (46.8)	3427 (42.1)
≥45	248 (8.2)	233 (9.9)	352 (9.3)	762 (9.4)
Rurality				
Urban	2599 (85.8)	1968 (83.8)	3239 (85.1)	6947 (85.3)
Rural	415 (13.7)	375 (16.0)	550 (14.5)	1161 (14.3)
Missing	14 (0.5)	6 (0.3)	15 (0.4)	32 (0.4)
Neighborhood income quintile				
1 (poorest)	1000 (33.0)	715 (30.4)	1185 (31.2)	2524 (31.0)
2	609 (20.1)	491 (20.9)	810 (21.3)	1699 (20.9)
3	553 (18.3)	438 (18.6)	658 (17.3)	1459 (17.9)
4	480 (15.9)	371 (15.8)	592 (15.6)	1303 (16.0)
5 (Richest)	372 (12.3)	327 (13.9)	543 (14.2)	1303 (16.0)
Missing	14 (0.5)	7 (0.3)	17 (0.4)	35 (0.4)
Substance use ED visit or hospitalization in past 2 y				
Any	358 (11.8)	346 (14.7)	575 (15.1)	960 (11.8)
Alcohol	106 (3.5)	93 (4.0)	145 (3.8)	288 (3.5)
Opioids	46 (1.5)	23 (1.0)	38 (1.0)	90 (1.1)
Oher	244 (8.0)	270 (11.5)	448 (11.8)	687 (8.4)
Mental health ED visit or hospitalization in past 2 y				
Any	520 (17.2)	406 (17.3)	639 (16.8)	1353 (16.6)
Anxiety disorder	327 (10.8)	247 (10.5)	379 (10.0)	827 (10.2)
Mood disorder	164 (5.4)	133 (5.7)	185 (4.9)	430 (5.3)
Schizophrenia or psychosis	30 (1.0)	22 (0.9)	42 (1.1)	86 (1.1)
Other	180 (5.9)	146 (6.2)	221 (5.8)	450 (5.5)
Outpatient substance use or mental health visits in past 2 y				
Family medicine or general practice	1537 (50.8)	1191 (50.7)	1960 (51.5)	4126 (50.7)
Psychiatry	571 (18.9)	465 (19.8)	740 (19.5)	1564 (19.2)
Vomiting visits before index visit, No.				
0	2098 (69.3)	1743 (74.2)	2742 (72.1)	6031 (74.1)
1	555 (18.3)	340 (14.5)	627 (16.5)	1315 (16.2)
2	198 (6.5)	138 (5.9)	227 (6.0)	445 (5.5)
3	88 (2.9)	55 (2.3)	106 (2.8)	184 (2.3)
≥4	89 (2.9)	73 (3.1)	102 (2.7)	165 (2.0)

Abbreviation: ED, emergency department.

^a^ Characteristics taken at the time of first visit in each period. The same individual can appear only once in each period but in multiple periods. Consequently, the number of individuals across the 3 periods is greater than the total number of individuals across all periods.

^b^ Defined as June 2014 to September 2018 (57 months).

^c^ Defined as October 2018 to February 2020 (17 months).

^d^ Defined as March 2020 to May 2021 (16 months), which overlapped with the COVID-19 pandemic.

Table 2 shows the total and mean rates of ED visits for CHS and all-cause vomiting. Overall, there were 12 866 CHS ED visits and 29 516 ED visits meeting the sensitive CHS definition. Across all periods, the rate of CHS ED visits was highest in individuals aged 19 to 24 years and those living in the lowest income quintile neighborhoods (Table 2). The proportion of all-cause vomiting visits owing to CHS increased 4.7-fold from pre-legalization (4265 visits [2.4%]) to during the commercialization period (5515 visits [11.3%]). Cannabis increased from the fifth most common identified codiagnosis with vomiting before legalization to the most common codiagnosis during the commercialization period (eAppendix in the Supplement).

**Table 2.** ED Visits for CHS in Ontario, Canada, by Period

Measure	Prelegalization, ^a^	Legalization^b^	Commercialization^c^
Total visits, No. (% all-cause vomiting meeting CHS criteria)			
All-cause vomiting, No.^d^	178 523	58 407	49 018
CHS^e^	4265 (2.4)	3086 (5.3)	5515 (11.3)
CHS sensitive definition^f^	11 444 (6.4)	7003 (12.0)	11 069 (22.6)
ED visits, monthly mean rate (SD), per 100 000 individuals			
All-cause vomiting^d^	25.8 (2.1)	27.1 (2.0)	23.9 (4.0)
CHS^e^	0.6 (0.3)	1.4 (0.2)	2.7 (0.5)
CHS sensitive definition^f^	1.6 (0.7)	3.2 (0.4)	5.4 (1.0)
CHS ED visits, monthly mean rate (SD), per 100 000 individuals^e^			
Men	0.7 (0.3)	1.5 (0.2)	2.5 (0.4)
Women	0.6 (0.3)	1.4 (0.2)	2.9 (0.7)
Age, y			
15-18	1.5 (0.9)	3.9 (1.3)	5.7 (1.6)
19-24	2.6 (1.3)	5.7 (0.6)	10.8 (2.2)
25-44	0.8 (0.5)	1.9 (0.4)	3.9 (0.9)
≥45	0.1 (0.1)	0.2 (0.1)	0.4 (0.2)
Neighborhood income Q1	1.0 (0.6)	2.3 (0.4)	4.4 (0.8)
Neighborhood income Q5	0.4 (0.3)	0.9 (0.3)	1.7 (0.5)
CHS ED visits resulting in admission to hospital, No. (%)^e^			
Incident visits	315 (10.4)	183 (7.8)	216 (5.7)
All visits	487 (11.4)	269 (8.7)	379 (6.9)
CTAS score of CHS ED visits, No. (%)^e^			
Median (IQR)	3 (3-3)	3 (2-3)	3 (2-3)
1 (resuscitation)	7 (0.2)	≤5 (0.1)	8 (0.1)
2 (emergent)	983 (23.0)	801 (26.0)	1372 (24.9)
3 (urgent)	2722 (63.8)	1827 (59.2)	3380 (61.3)
4 (less urgent)	504 (11.8)	367 (11.9)	600 (10.9)
5 (non-urgent)	46 (1.1)	87 (2.8)	150 (2.7)
Missing	≤5 (0.1)	≤5 (0.1)	≤5 (0.1)
Recurrent CHS ED visits in 6 mo after incident visit, No. (%)^b^			
0	2639 (87.2)	1969 (83.8)	1852 (78.1)
1	293 (9.7)	272 (11.6)	323 (13.6)
2	67 (2.2)	63 (2.7)	117 (4.9)
3	19 (0.6)	26 (1.1)	42 (1.8)
≥4	10 (0.3)	19 (0.8)	37 (1.6)

Abbreviations: CHS, cannabis hyperemesis syndrome; ED, emergency department.

^a^ Defined as June 2014 to September 2018 (57 months).

^b^ Defined as October 2018 to February 2020 (17 months).

^c^ Defined as March 2020 to May 2021 (16 months), which overlapped with the COVID-19 pandemic.

^d^ Defined as *International Statistical Classification of Diseases and Related Health Problems, Tenth Revision *(*ICD-10-CA*) code R11 as the most responsible diagnosis.

^e^ Defined as *ICD-10-CA* code R11 as the most responsible diagnosis with a codiagnosis of code F12 or T40.7.

^f^ Defined as *ICD-10-CA* code R11 as the most responsible diagnosis with a codiagnosis of code F12 or T40.7 or with another ED visit with a diagnosis code F12 or T40.7 in the 6 months before or after the vomiting (R11) ED visit.

Across all periods, 11 085 of 12 866 CHS ED visits (86.1%) were classified emergent or urgent. The number of visits leading to hospitalization during an incident CHS ED visit decreased from 487 individuals (11.4%) before legalization to 379 individuals (6.9%) during the commercialization period. The number of individuals with 1 or more repeat CHS ED visits in the 6 months after their initial CHS visit increased from 389 individuals (12.8%) before legalization to 519 individuals (21.9%) during the commercialization period.

**Figure 1.**
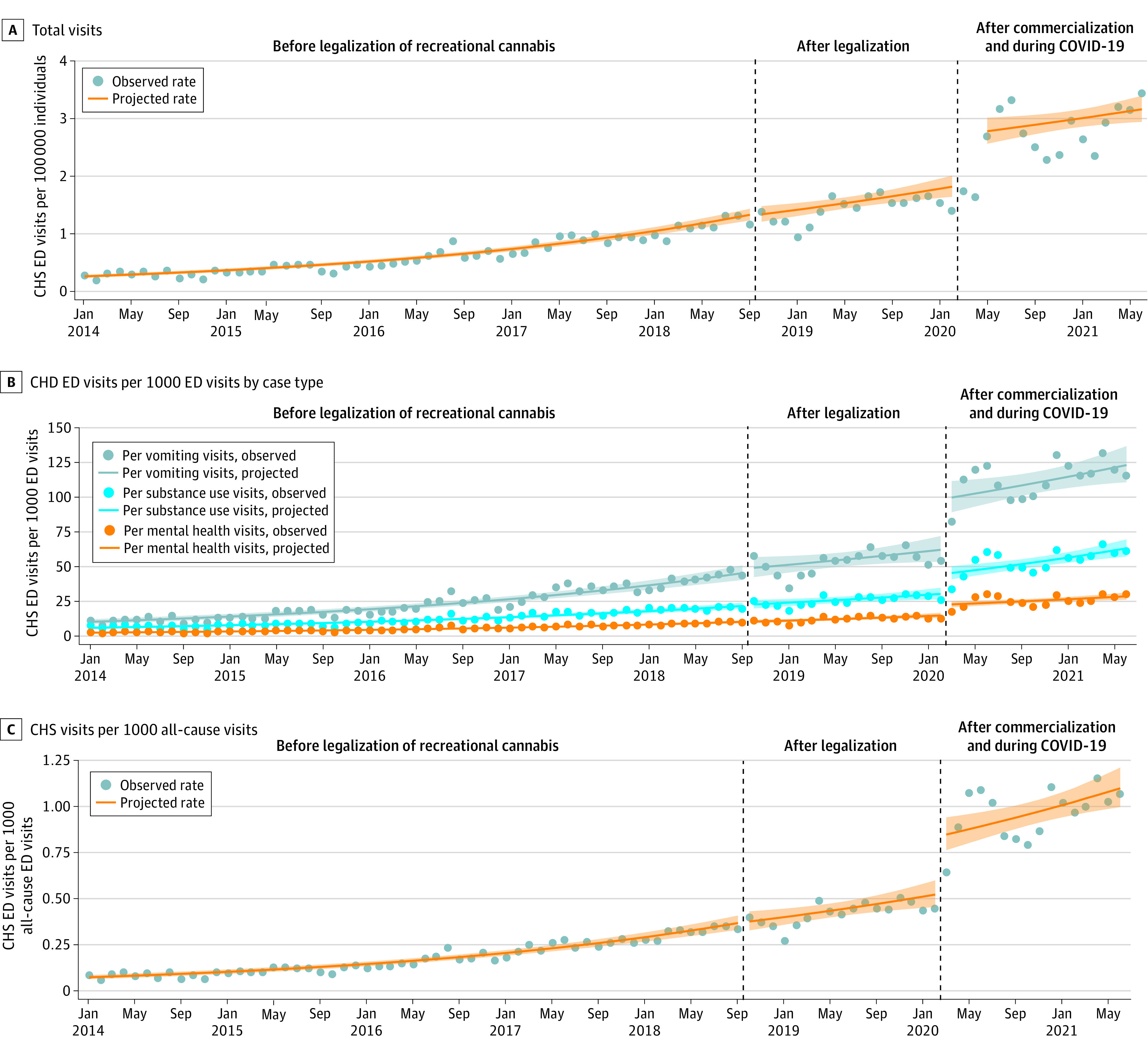
Observed and Projected Rates of Cannabis Hyperemesis Syndrome (CHS) Emergency Department (ED) Visits During Different Phases of Recreational Cannabis Legalization in Ontario, Canada Shaded regions indicate 95% CIs.

Figure 1 displays rates of CHS ED visits between January 2014 and June 2021 per 100 000 individuals and per 100 all-cause vomiting, substance use, mental health, or total ED visits. The monthly rate of CHS ED visits among individuals aged at least 15 years was 0.26 visits per 100 000 individuals in January 2014, 1.16 visits per 100 000 individuals in September 2018, 1.38 visits per 100 000 individuals in February 2020, and 3.40 visits per 100 000 individuals in June 2021.

Prior to legalization, CHS ED visits were increasing over time. Legalization was not associated with an immediate (intercept) or gradual (slope) change in rates of visits per capita or for any subtype of ED visits. After commercialization and at the onset of the COVID-19 pandemic, there was a large immediate increase in CHS visits per capita and per other types of ED visits. Compared with the counterfactual (had commercialization and COVID-19 not occurred), the combined change after 16 months was a significant net increase of 32% in the rate of CHS ED visits per capita (IRR, 1.32; 95% CI, 1.07-1.63) and a significant net increase of 57% in the rate of CHS ED visits per all-cause vomiting visits (IRR, 1.57; 95% CI, 1.13-2.18). Table 3 reports the segmented regression coefficients for the level and slope change for each period for the other outcomes.

**Table 3.** Overall Relative Changes in Rates of Monthly CHS ED Visits Per Capita and Per All Visits in Ontario, Canada, Following Recreational Cannabis Legalization and Commercialization and the COVID-19 Pandemic

Measure	CHS visits, incidence rate ratio (95% CI)				
	Per capita^a^	Per all-cause ED visits	Per all-cause vomiting visits	Per mental health visits	Per substance use visits
Prelegalization monthly slope	1.03 (1.03-1.03)	1.03 (1.03-1.03)	1.03 (1.02-1.03)	1.02 (1.02-1.03)	1.03 (1.02-1.03)
Legalization period					
Immediate change	0.99 (0.87-1.12)	1.00 (0.85-1.18)	1.07 (0.90-1.28)	1.03 (0.88-1.22)	1.00 (0.85-1.16)
Gradual change	0.99 (0.98-1.00)	0.99 (0.98-1.01)	0.99 (0.97-1.00)	0.99 (0.98-1.01)	1.00 (0.98-1.01)
Monthly slope	1.02 (1.01-1.03)	1.02 (1.01-1.03)	1.01 (1.00-1.03)	1.02 (1.00-1.03)	1.02 (1.01-1.04)
Net change 17 mo after legalization^b^	0.83 (0.68-1.02)	0.87 (0.66-1.15)	0.88 (0.65-1.19)	0.92 (0.71-1.19)	0.94 (0.72-1.25)
Commercialization and COVID-19					
Immediate change	1.49 (1.31-1.70)	1.60 (1.37-1.87)	1.59 (1.34-1.87)	1.47 (1.26-1.72)	1.53 (1.32-1.77)
Gradual change	0.99 (0.98-1.00)	1.00 (0.98-1.01)	1.00 (0.98-1.02)	1.00 (0.99-1.02)	0.99 (0.98-1.01)
Monthly slope	1.01 (1.00-1.02)	1.02 (1.01-1.03)	1.01 (1.00-1.03)	1.02 (1.01-1.03)	1.02 (1.01-1.03)
Net change 16 mo after commercialization and COVID-19^c^	1.32 (1.07-1.63)	1.52 (1.12-2.06)	1.57 (1.13-2.18)	1.38 (1.04-1.82)	1.56 (1.15-2.11)

Abbreviations: CHS, cannabis hyperemesis syndrome; ED, emergency department.

^a^ Per capita visits include adjustment for March and April 2020, when all-cause ED visits declined during first months of COVID-19 pandemic.

^b^ Relative to the counterfactual secular trend from the prelegalization period.

^c^ Relative to the counterfactual trend from the legalization with strict control period.

**Figure 2.**
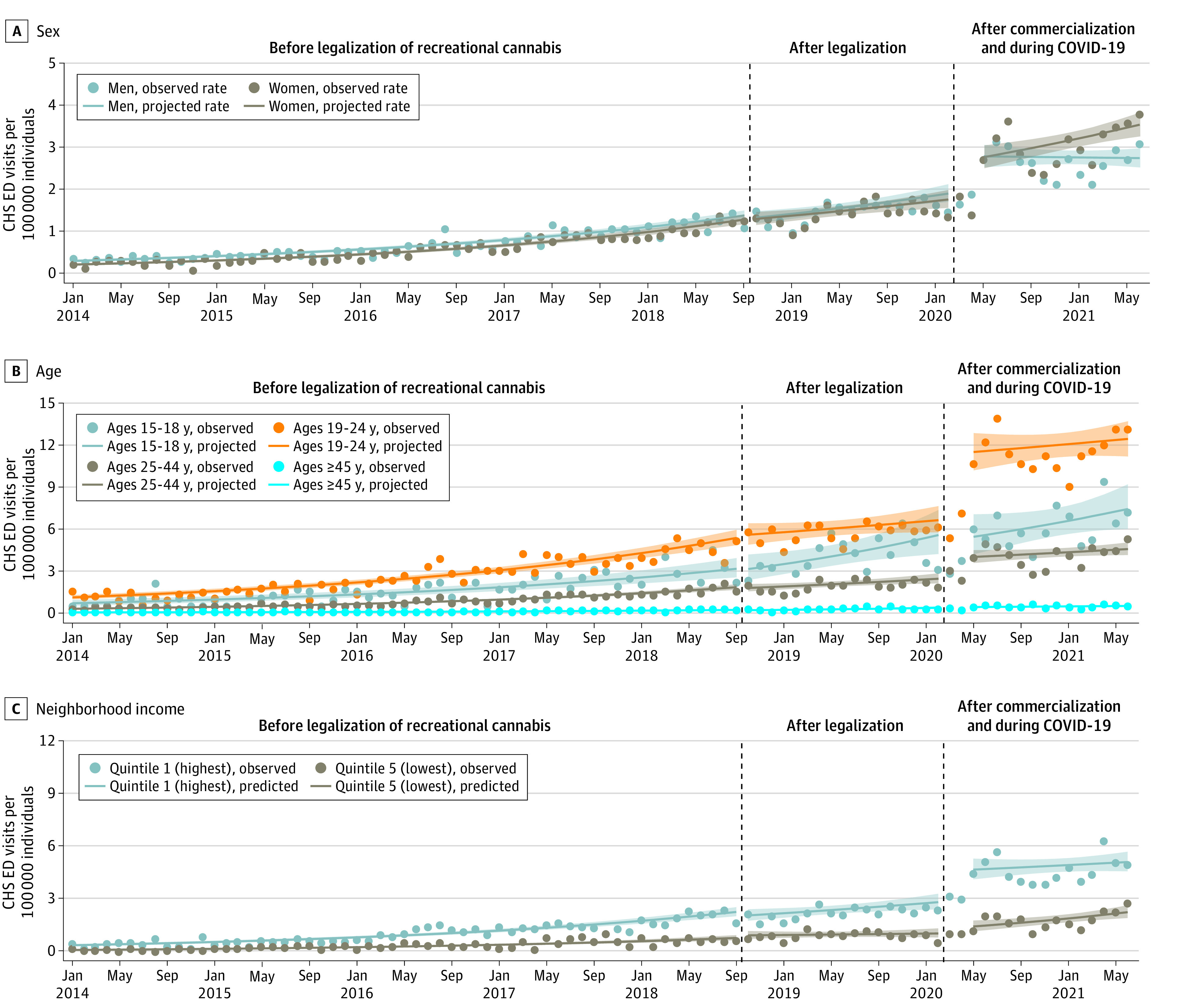
Observed and Projected Rates of Cannabis Hyperemesis Syndrome (CHS) Emergency Department (ED) Visits Per 100 000 Individuals During Different Phases of Recreational Cannabis Legalization in Ontario, Canada Shaded regions indicate 95% CIs.

Figure 2 shows the subgroup CHS ED visit rates per 100 000 individuals between January 2014 and June 2021. Legalization was not associated with an immediate or gradual changes in rates of visits per capita in any subgroup. Compared with the counterfactual, commercialization was associated with a net increase in rates of CHS ED visits per capita for individuals aged 19 to 24 years (IRR, 1.60; 95% CI, 1.19-2.16) and individuals aged 25 to 44 years (IRR, 1.40; 95% CI, 1.04-1.90) but not with net increases for individuals aged 15 to 18 years (IRR, 0.78; 95% CI, 0.42-1.45) or 45 years and older (IRR, 0.77; 95% CI, 0.43-1.36). Compared with the counterfactual, commercialization was associated with a net increase of 49% in rates of CHS ED visits per capita for women (IRR, 1.49; 95% CI, 1.16-1.92) but not with net increases for men (IRR, 1.08; 95% CI, 0.85-1.37). Finally, commercialization was associated with an immediate increase in visits for individuals living in the lowest income quintile but not for those living in the highest income quintile neighborhoods (eTable 2 and eTable 3 in the Supplement).

## Discussion

To our knowledge, this repeated cross-sectional study is the largest study to date examining the characteristics of individuals presenting to the ED for CHS and how such visits changed after the legalization of cannabis. Over 7.5 years, the rates of CHS ED visits increased 13-fold (from 0.26 visits per 100 000 individuals in January 2015 to 3.40 visits per 100 000 individuals in June 2021). CHS ED visits were increasing over time before legalization, and this trend continued after legalization with restricted retail stores and products. However, during the period that the cannabis retail market in Ontario commercialized with new cannabis products and unlimited stores, which overlapped with the COVID-19 pandemic, CHS ED visits increased substantially.

The overlap of the commercialization of Ontario’s cannabis retail market and the COVID-19 pandemic challenges attribution of each event to the observed increases in CHS ED visits. However, 3 points support that commercialization likely played a role. First, we observed that increases in CHS ED visits during the pandemic were much larger than changes in ED visits for mental health and substance use. These findings suggest that increases in cannabis harms were associated with a factor other than deteriorating mental health and increased substance use during the pandemic. Second, we did not observe an increase in CHS ED visits for individuals younger than the legal age of cannabis purchase in Ontario, but we did observe large increases among young adults who could access the legal market. This group of young adults arguably have faced similar mental health and social challenges during the pandemic (eg, social isolation and school interruptions) and experienced similar changes in patterns of ED visits for mental health and addictions in Ontario.^18,25^ These findings suggest that greater access to a commercial cannabis market may be driving increases in CHS visits. Third, the risk factor for CHS is regular use of high-potency cannabis. Sales data supports that the strength (THC content) of cannabis products has increased substantially since legalization, particularly when new products were introduced during commercialization.^26,27^ Prior work has also found that increases in cannabis sales during the pandemic were better explained my market evolution rather than COVID-19 factors, further supporting a commercialization role.^28^

Our findings have important policy implications within Canada and for other jurisdictions that have already or are considered legalizing recreational cannabis. While prior studies have broadly found that legalization is associated with increases in CHS presentations, our results suggest that regulatory approaches may influence these changes.^9,10,11,12^ We found that legalization with strict controls over retail store access and product type was not associated with large changes in CHS. In contrast, allowing the commercialization of recreational cannabis may be associated with substantial increases in heavy and regular cannabis use, leading to CHS. Collectively, these findings suggest that policies that restrict product variety and strength and place limits on retail store access may reduce increases in CHS after legalization. In addition, our observation that CHS ED visits increased for individuals older than but not younger than the legal age of purchase suggests that setting a higher legal age of purchase could reduce harms from legalization in youth and young adults.

This is the largest study to our knowledge to describe the characteristics and clinical outcomes of individuals with CHS. We found that CHS ED visits were highest in young adults and individuals living in low-income neighborhoods and occurred equally in men and women, which is consistent with patterns of self-reported daily cannabis use.^2,29^ We observed that women and young adults (older than the legal purchase age) experienced the largest increases in CHS ED visits after legalization. These findings suggest that changes in harmful cannabis use after commercialization may differ substantially by subgroups, which should be considered in policy decisions. Additionally, this highlights the need for further research understand drivers of these trends, in addition to targeted preventive efforts.

Our findings also potentially have immediate clinical implications. More than three-quarters of individuals with CHS ED visits had no prior mental health– or substance-related ED visits or hospitalizations in the past 2 years. CHS ED visits in our study presented as high acuity events, with 86% of CHS visits triaged in the 3 most urgent categories compared with 61% of all-cause ED visits in 2017.^30^ In addition, a substantial proportion of individuals experienced a repeat CHS ED visit in the 6 months after their initial CHS visit. These findings suggest that ED visits for CHS may be an important and burdensome first point of contact with the acute health service. Better recognition of CHS symptoms by ED staff could allow more timely diagnosis and engagement with appropriate treatment to reduce or cease cannabis use.

### Limitations

Our study has several limitations. First, there is currently no diagnostic code for CHS, and our study infers CHS from a combination of diagnostic codes previously used in the literature.^9,10^ Importantly, the specificity and sensitivity of this coding combination are currently unknown, and our study may underestimate the burden of CHS, which is often not considered as a cause of vomiting in ED visits.^19^ Second, the observed increases could be from a greater awareness of CHS by ED physicians or the willingness of patients to disclose cannabis use. Importantly, while both possibilities could explain changes immediately after legalization, they are far less likely to be responsible for the large increase observed beginning 18 months after legalization. Third, as previously mentioned, the overlap of commercialization and the COVID-19 pandemic limits our ability to separate the relative contributions of each event. Fourth, the retail market has continued to expand since the end of our study, and our results may not capture the health impacts of a fully mature commercialized market.

## Conclusions

The findings of this cross-sectional study suggest that, while prevalence of CHS is expected to increase after legalization, commercialization of cannabis, by allowing access to retail stores and higher potency products, may be associated with even larger increases. When unrecognized, CHS can generate significant morbidity and treatment costs. Greater awareness of symptoms of CHS by physicians in regions with legal recreational and particularly commercial cannabis markets is indicated.

## Notes

Supplement. **eMethods.** Data Sources Information and Covariate Details **eAppendix.** Sensitivity Analysis **eTable 1.** Five Most Commonly Occurring Codiagnoses for ED Visits Owing to Vomiting (*ICD-10-CA* Code R11) by Time Period **eTable 2.** Changes in Rates of Monthly CHS ED Visits Per Capita for Subgroups Following Recreational Cannabis Legalization and Commercialization and COVID-19 **eTable 3.** Changes in Rates of Monthly CHS ED Visits Per Capita for Subgroups Following Recreational Cannabis Legalization and Commercialization and COVID-19
