# Supplementary material for: Changes in Emergency Department Visits for Cannabis Hyperemesis Syndrome Following Recreational Cannabis Legalization and Subsequent Commercialization in Ontario, Canada
Source: JAMA Netw Open. 2022 Sep 16;5(9):e2231937. doi: 10.1001/jamanetworkopen.2022.31937 (PMC9482056; doi:10.1001/jamanetworkopen.2022.31937)
Supplement: Supplement. — eMethods. Data Sources Information and Covariate Details eAppendix. Sensitivity Analysis eTable 1. Five Most Commonly Occurring Codiagnoses for ED Visits Owing to Vomiting (ICD-10-CA Code R11) by Time Period eTable 2. Changes in Rates of Monthly CHS ED Visits Per Capita for Subgroups Following Recreational Cannabis Legalization and Commercialization and COVID-19 eTable 3. Changes in Rates of Monthly CHS ED Visits Per Capita for Subgroups Following Recreational Cannabis Legalization and Commercialization and COVID-19 [file jamanetwopen-e2231937-s001.pdf]

Supplemental Online Content

Myran DT, Roberts R, Pugliese M, Taljaard M, Tanuseputro P, Pacula RL. Changes in emergency department visits for cannabis hyperemesis syndrome following recreational cannabis legalization and subsequent commercialization in Ontario, Canada. *JAMA Netw Open*. 2022;5(9):e2231937. doi:10.1001/jamanetworkopen.2022.31937

eMethods. Data Sources Information and Covariate Details
eAppendix. Sensitivity Analysis
eTable 1. Five Most Commonly Occurring Codiagnoses for ED Visits Owing to Vomiting (*ICD-10-CA* Code R11) by Time Period
eTable 2. Changes in Rates of Monthly CHS ED Visits Per Capita for Subgroups Following Recreational Cannabis Legalization and Commercialization and COVID-19
eTable 3. Changes in Rates of Monthly CHS ED Visits Per Capita for Subgroups Following Recreational Cannabis Legalization and Commercialization and COVID-19

This supplemental material has been provided by the authors to give readers additional information about their work.

© 2022 Myran DT et al. *JAMA Network Open.*

eMethods. Data Sources Information and Covariate Details

Data Sources Information

Linked Datasets Used in Study

We obtained study data from de-identified and linked health administrative databases housed at ICES. ICES is an independent, non-profit research institute funded by an annual grant from the Ontario Ministry of Health and Long-Term Care (MOHLTC). As a prescribed entity under Ontario's privacy legislation, ICES is authorized to collect and use health care data for the purposes of health system analysis, evaluation and decision support. Secure access to these data is governed by policies and procedures that are approved by the Information and Privacy Commissioner of Ontario. In 2018, the institute formerly known as the Institute for Clinical Evaluative Sciences formally adopted the initialism ICES as its official name.

The dataset from this study is held securely in coded form at ICES. While legal data sharing agreements between ICES and data providers (e.g., healthcare organizations and government) prohibit ICES from making the dataset publicly available, access may be granted to those who meet pre-specified criteria for confidential access, available at www.ices.on.ca/DAS (email: das@ices.on.ca). The full dataset creation plan and underlying analytic code are available from the authors upon request, understanding that the computer programs may rely upon coding templates or macros that are unique to ICES and are therefore either inaccessible or may require modification.

These datasets were linked using unique encoded identifiers and analyzed at ICES.
We used the following databases:

- National Ambulatory Care Reporting System (NACRS), which captures all ED visits and the cause of the visit within Ontario;
- Discharge Abstract Database (DAD), which includes records for all acute care hospitalizations in Ontario,
- Ontario Mental Health Reporting System Metadata (OMHRS) which includes all mental health hospitalizations in Ontario,
- OHIP Claims Database (OHIP, which captures all outpatient visits (including virtual) and the reason for visit in Ontario;
- Registered Persons Database (RPDB), which includes the total number of persons at-risk each month and individuals' age and sex; and
- Postal Code Conversation File+ (PCCF+)which contains information on the rurality (urban vs rural) and neighbourhood income for each person's home address.

These datasets were linked using unique encoded identifiers and analyzed at ICES.

© 2022 Myran DT et al. *JAMA Network Open.*

Exposure, Outcomes and Additional Covariates

Type	Name	Database	Codes/Description
Exposure	Period	NA	Calendar Dates<br>Pre-legalization: January 2014 – September 2018<br>Legalization: October 2018 – February 2020<br>Commercialization/COVID: March 2020 – May 2021
Descriptor	Age	RPDB	Age at index (an individuals first CHS specific ED visit in each period)
Descriptor	Sex	RPDB	Sex at index (an individuals first CHS specific ED visit in each period)
Descriptor	Rurality	RPDB; PCCF+	We used Statistics Canada's definition for rurality, which defines a rural residence as living in a town or municipality outside of a census metropolitan areas (CMA, population of 100,000 or more) or census agglomeration (CA, population of 10,000 or more).(1)
Descriptor	Neighbourhood Income Quintile	RPDB; PCCF+	We classified individuals' neighbourhoods into income quintiles based on the average before-tax income, adjusted for household size and relative to other neighbourhoods either within the same CMA or CA or in rural Ontario.(2)
Descriptor	Substance Use ED visit or Hospitalization in Past 2 Years	NACRS; DAD; OMHRS	Substance Use ED visit or Hospitalization within 2 years before index (an individuals first CHS specific ED visit in each period)<br>Substance Use Codes from: Mental Health and Addictions Scorecard and Evaluation Framework indicator.(3)
Descriptor	Mental Health ED visit or Hospitalization in Past 2 Years	NACRS; DAD; OMHRS	Mental Health ED visit or Hospitalization within 2 years before index (an individuals first CHS specific ED visit in each period)<br>Mental Health Codes from: Mental Health and Addictions Scorecard and Evaluation Framework indicator.(3)
Descriptor	Outpatient Substance Use or Mental health Visits in Past 2 Years	OHIP	Substance Use or Mental Health outpatient visit within 2 years before index (an individuals first CHS specific ED visit in each period)<br>Codes from: Mental Health and Addictions Scorecard and Evaluation Framework indicator.(3)

© 2022 Myran DT et al. *JAMA Network Open.*

Type	Name	Database	Codes/Description
Descriptor	Vomiting Visits Before Index Visit	NACRS	ED visit with ICD-10-CA code R11
Outcome	CHS specific ED visit	NACRS	ED visit with ICD-10-CA code R11 + F12 or T40.7, same ED visit
Outcome	CHS sensitive ED visit	NACRS	ICD-10-CA code R11 + F12 or T40.7 ED in same visit or another ED visit +/- 6 months
Outcome	All-cause ED visits	NACRS	All ED visits regardless of ICD-10-CA code
Outcome	Vomiting ED visit	NACRS	ED visit with ICD-10-CA code R11
Outcome	Mental Health ED Visit	NACRS	Mental Health Codes from: Mental Health and Addictions Scorecard and Evaluation Framework indicator.(3)
Outcome	Substance Use ED visit	NACRS	Substance Use Codes from: Mental Health and Addictions Scorecard and Evaluation Framework indicator.(3)
Outcome	CHS specific ED visits resulting in Admission to Hospital	NACRS, DAD, OMHRS	
Outcome	CTAS	NACRS	Canadian Trauma and Acuity Scale (CTAS)(4)
Outcome	Recurrent CHS specific ED visits in 6 months after incident visit	NACRS	ED visit with ICD-10-CA code R11 + F12 or T40.7, same ED visit

Statistical Analysis

When describing period specific characteristics each individual can only contribute characteristics once per period but could contribute in multiple periods. When describing the whole study period characteristics individuals could only contribute once per study with characteristics obtained at the time of first visit.

© 2022 Myran DT et al. *JAMA Network Open.*

eAppendix. Sensitivity Analysis

We examined the five most common co-diagnoses for vomiting visits across the three periods. Pre-legalization, just under half (47.9%) of all vomiting visits had at least one co-diagnosis and a cannabis harm was the 5th most common, involved in 2.2% of all vomiting cases. Cannabis harms increased to the 4th most common diagnosis during the RCL period and the 2nd most common co-diagnosis during the RCC period, involved in 11.1% of all vomiting cases, while the share of vomiting visits with a co-diagnosis rose slightly overall, to 52.9% in the RCC period. Unspecified gastroenteritis, which was the most common co-diagnosis prior to legalization at 10.4% fell to the fourth most common co-diagnosis in the RCC period, involved in just 6.4% of all vomiting cases. See supplement Table 1 for details.

1. Statistics Canada. Population Centre and Rural Area Classification 2016. 2016.
2. Canadian Institute for Health Information. Measuring Health Inequalities: A ToolkitArea-Level Equity Stratifiers Using PCCF and PCCF+. 2018.
3. MHASEF Research Team. Mental Health and Addictions System Performance in Ontario: A Baseline Scorecard [Internet]. Toronto; 2018 [cited 2022 Mar 21]. Available from: https://www.ices.on.ca/Publications/Atlases-and-Reports/2018/MHASEF
4. Gravel J GSGR et al. The Canadian Triage and Acuity Scale for Children: A Prospective Multicenter Evaluation. Annals of Emergency Medicine [Internet]. 2012 [cited 2022 Mar 20];60(1):71-77.e3. Available from: doi:10.1016/J.ANNEMERGMED.2011.12.004

© 2022 Myran DT et al. *JAMA Network Open.*

eTable 1. Five Most Commonly Occurring Codiagnoses for ED Visits Owing to Vomiting (ICD-10-CA Code R11) by Time Period

	Pre-Legalization		Recreational Cannabis Legalization		Recreational Cannabis Commercialization/COVID-19	
	Jan 2014 – Sep 2018 (57 months)		Oct 2018 – Feb 2020 (17 months)		Mar 2020 – May 2021 (13 months)	
	N = 178,526		N = 58,410		N = 49,036	
Rank	Co-diagnosis (ICD)	N (%)	Co-diagnosis (ICD)	N (%)	Co-diagnosis (ICD)	N (%)
1	None	93,070 (52.1)	None	30,048 (51.4)	None	23,089 (47.1)
2	Gastroenteritis, unspecified (A099)	18,640 (10.4)	Gastroenteritis, unspecified (A099)	5,582 (9.6)	**Cannabis (F12, T40.7)**	5,525 (11.3)
3	Diabetes mellitus without complications (E109, E119, E149)	12,587 (7.1)	Diabetes mellitus without complications (E109, E119, E149)	4,272 (7.3)	Diabetes mellitus without complications (E109, E119, E149)	3,636 (7.40)
4	Other and unspecified abdominal pain (R104)	4783 (2.7)	**Cannabis (F12, T40.7)**	3,093 (5.3)	Gastroenteritis, unspecified (A099)	3,146 (6.4)
5	**Cannabis (F12, T40.7)**	4,272 (2.4)	Other and unspecified abdominal pain (R104)	1,703 (2.9)	Other and unspecified abdominal pain (R104)	1,633 (3.3)

Percentages are column percentages.

© 2022 Myran DT et al. *JAMA Network Open.*

eTable 2. Changes in Rates of Monthly CHS ED Visits Per Capita[a] for Subgroups Following Recreational Cannabis Legalization and Commercialization and COVID-19

	CHS Visits Per Capita[a]			
	Age 15-18 y	Aged 19-24 y	Aged 25-44 y	Aged 45+ y
	Incidence Rate Ratio (95% CI)			
Pre-Legalization Monthly Slope[a]	1.03 (1.02 - 1.04)	1.03 (1.03 - 1.03)	1.03 (1.03 - 1.04)	1.04 (1.03 - 1.05)
Legalization Immediate Change	0.96 (0.68 - 1.36)	1.03 (0.87 - 1.22)	0.95 (0.80 - 1.14)	0.86 (0.61 - 1.21)
Legalization Gradual Change	1.01 (0.98 - 1.04)	0.98 (0.97 - 1.00)	0.99 (0.97 - 1.00)	1.00 (0.98 - 1.03)
Post Legalization Monthly Slope	1.04 (1.01 - 1.06)	1.01 (1.00 - 1.02)	1.02 (1.01 - 1.03)	1.04 (1.02 - 1.07)
**Net Change 17 Months After Legalization[b]**	1.10 (0.63 - 1.93)	0.78 (0.59 – 1.02)	0.77 (0.57-1.03)	0.92 (0.53-1.59)
Commercialization/ COVID-19 Immediate Change	0.91 (0.62 - 1.33)	1.71 (1.42 – 2.04)	1.59 (1.33 - 1.91)	1.11 (0.79 - 1.56)
Commercialization/ COVID-19 Gradual Change	0.99 (0.95 - 1.03)	1.00 (0.98 - 1.01)	0.99 (0.97 - 1.01)	0.97 (0.94 - 1.01)
Post Commercialization/ COVID-19 Monthly Slope	1.02 (1.00 - 1.05)	1.01 (0.99 - 1.02)	1.01 (1.00 - 1.02)	1.01 (0.99 - 1.04)
**Net Change 16 Months after Commercialization/COVID-19[c]**	0.78 (0.42 – 1.45)	1.60 (1.19 – 2.16)	1.40 (1.04-1.90)	0.77 (0.43-1.36)

[a]Per Capita visits include adjustment for March and April 2020 when all-cause ED visits declined during first months of COVID-19 pandemic
[b]Relative to the counterfactual secular trend from the pre-legalization period
[c]Relative to the counterfactual trend from the legalization with strict control period

© 2022 Myran DT et al. *JAMA Network Open.*

eTable 3. Changes in Rates of Monthly CHS ED Visits Per Capita for Subgroups Following Recreational Cannabis Legalization and Commercialization and COVID-19

	CHS Visits Per Capita[a]			
	Men	Women	Lowest (poorest) Income Quintile	Highest (richest income quintile)
	Incidence Rate Ratio (95% CI)			
Pre-Legalization Monthly Slope[a]	1.03 (1.03 - 1.03)	1.03 (1.03 - 1.04)	1.03 (1.03 - 1.04)	1.03 (1.03 - 1.04)
Legalization Immediate Change	0.94 (0.82 - 1.07)	0.99 (0.86 - 1.15)	0.90 (0.75 - 1.08)	1.17 (0.89 - 1.55)
Legalization Gradual Change	0.99 (0.98 - 1.01)	0.99 (0.97 - 1.00)	0.99 (0.97 - 1.00)	0.97 (0.95 - 1.00)
Post Legalization Monthly Slope	1.02 (1.01 - 1.03)	1.02 (1.01 - 1.03)	1.02 (1.00 - 1.03)	1.01 (0.98 - 1.03)
**Net Change 17 Months After Legalization[b]**	0.86 (0.69 - 1.08)	0.80 (0.63 - 1.02)	0.74 (0.55 -1.01)	0.78 (0.49 -1.23)
Commercialization/COVID-19 Immediate Change	1.47 (1.28 - 1.70)	1.49 (1.28 - 1.73)	1.62 (1.33 - 1.97)	1.24 (0.92 – 1.68)
Commercialization/COVID-19 Gradual Change	0.98 (0.96 - 0.99)	1.00 (0.98 - 1.02)	0.99 (0.97 - 1.01)	1.03 (1.00 - 1.06)
Post Commercialization/COVID-19 Monthly Slope	1.00 (0.99 - 1.01)	1.02 (1.01 - 1.03)	1.01 (0.99 - 1.02)	1.03 (1.01 - 1.06)
**Net Change 16 Months after Commercialization/COVID-19[c]**	1.08 (0.85 - 1.37)	1.49 (1.16 - 1.92)	1.38 (0.99 -1.90)	1.77 (1.07 -2.90)

[a]Per Capita visits include adjustment for March and April 2020 when all-cause ED visits declined during first months of COVID-19 pandemic
[b]Relative to the counterfactual secular trend from the pre-legalization period
[c]Relative to the counterfactual trend from the legalization with strict control period

© 2022 Myran DT et al. *JAMA Network Open.*
